# Science and Ethics in Epidemiology

**DOI:** 10.2188/jea.14.105

**Published:** 2005-03-18

**Authors:** Shigeru Hisamichi

**Affiliations:** 1 Division of Epidemiology, Department of Public Health and Forensic Medicine, Tohoku University Graduate School of Medicine.

**Keywords:** epidemiology, science, ethics

## Abstract

This article is the summary of my Special Lecture at the 14th Annual Scientific Meeting of the Japan Epidemiological Association in 2004. Epidemiology is defined as the “science of investigating the distribution of diseases in human populations and their determinants.” Recent advances in study methodology, especially a widespread conduct of randomized controlled trials (RCTs), have strengthened the scientific basis of epidemiology. When a highly scientific method of investigation is applied to humans, the ethical aspects of the study also become an issue. However, it would also be unethical to use new drugs and vaccines without scientific evidence. The ethical aspects and the scientific aspects of epidemiologic research are thus both very important, but conflict with each other, often causing dilemmas. I would discuss how we could solve these dilemmas and thus contribute ourselves to health promotion and disease prevention of human populations. Finally, I would propose the new paradigm of changing epidemiology into a “neotype science” and transformation of EKIGAKU (epidemiology) into EKIGAKU (beneficial science).

## Introduction

Epidemiology is defined as the “science of investigating the distribution of diseases in human populations and their determinants.” It is only natural that human beings—both patients and healthy people—become the targets of such analyses. Epidemiology has the following four objectives: (1) to elucidate the natural history of diseases, (2) to determine the presence of disease epidemics, (3) to collect information regarding the distribution and causes of diseases, and finally (4) to search for keys to the causes of diseases.

Epidemiologic study can be classified into observational epidemiology and interventional epidemiology, and the former can be further subdivided into descriptive epidemiology and analytical epidemiology. Observational epidemiology deals with setting hypotheses for the question, “What is the cause of the disease?” and then a search for the key to various events that occur in human populations using various techniques. The causal relationship is then confirmed by interventional epidemiologic study, otherwise called experimental epidemiology. Clinical studies to investigate the efficacy of new drugs and field studies to evaluate the effects of vaccines are some examples of experimental epidemiology.

## Is EKIGAKU (‘epidemiology’) EKIGAKU (‘fortune telling’)?

This comparison is often made in Japan, because the Japanese words for “epidemiology” and “fortune telling” are pronounced in the same way (“EKIGAKU”). Unfortunately, some people in Japan regard epidemiology as “a kind of fortune telling, with a fifty-fifty probability.” There seems to be a misunderstanding among the general public regarding the scientific nature of epidemiology, and, in this context, epidemiologists cannot be completely spared the responsibility for promoting such a misunderstanding. Some people still believe that epidemiologic studies are merely a presentation of statistical data and are not part of the scientific main stream. However, recent advances in epidemiologic study techniques have demonstrated that epidemiology is far from being unscientific. Here, I introduce two events that have attracted people’s attention in recent years.

## Does green tea inhibit the development of gastric cancer?

There have been numerous studies of the effects of green tea in preventing cancer. Basic biochemical studies and animal experiments have reported that the catechins responsible for the astringent taste of green tea possess cancer-inhibitory activity. Most case-control studies have reported that green tea consumption reduces the risk of death from gastric cancer (Table 1).^1^^-^^8^ However, most prospective cohort studies have not shown that drinking green tea reduces the risk of gastric cancer (Table 2).^9^^-^^14^

**Table 1.** Case-control studies of green tea and gastric cancer*

Author (Year)	Country	No. of cases	No. of controls	Type of controls	Results		Conclusions
Tajima et al. (1985)^1^	Japan (Aichi)	93	186	Hospital	OR for 4+ vs. <4 cups/d		Protective
					0.64(NS)		NS

Kono et al. (1988)^2^	Japan (Saga)	139	2,574	Hospital	OR (95%CI) for 10+ vs. <10 cups/d		Protective
			278	Population	0.5 (0.3-1.1) as compared with hospital controls		Significant
					0.3 (0.1-0.7) as compared with population controls		

Lee et al. (1990)^3^	Taiwan	210	810	Hospital	OR for drinkers vs. nondrinkers		Increased risk
					2.00 (P < 0.10)		NS

Yu et al. (1991)^4^	China (Shanghai)	84	2,676	Population	OR (95%CI) for users vs. nonusers		Protective
					0.3 (0.1-0.7)		Significant

Yu et al. (1995)^5^	China (Shanghai)	711	711	Population	OR (95%CI) for drinkers vs. nondrinkers		Protective
					0.73 (0.55-0.97)		Significant

Ji et al. (1996)^6^	China (Shanghai)	1,124	1,451	Population	OR (95%CI) for regular drinkers vs. nondrinkers		Protective
					Men	0.96(0.77-1.21)	NS
					Women	0.77(0.52-1.13)	

Inoue et al. (1998)^7^	Japan (Aichi)	893	21,128	Hospital	OR (95%CI)		Protecitve
					Rarely	1.00(Reference)	Significant
					Occasionally	1.00(0.77-1.44)	
					1-3 cups/d	0.96(0.70-1.32)	
					4-6 cups/d	1.01(0.74-1.39)	
					7+ cups/d	0.69(0.48-1.00)	

Setiawan et al. (2001)^8^	China (Yangzhong)	132	433	Population	OR (95%CI)		Protective
					Nondrinkers	1.00(Reference)	Significant
					1-21 cups/wk	0.70(0.36-1.36)	
					22+ cups/wk	0.39(0.15-1.01)	
					Trend P = 0.0479		

* NS, not significant. OR, odds ratio. CI, confidence interval.

**Table 2.** Prospective studies of green tea and gastric cancer*

Author (Year)	Country	No. of cases	No. of noncases	Results		Conclusions
Galanis et al. (1998)^9^	Japanese in Hawaii	108	11,799	RR (95%CI) according to no. of cups/d		Increased risk
				0	1.0(Reference)	NS
				1	1.3(0.7-2.1)	
				2+	1.5(0.9-2.3)	
				Trend P = 0.10		

Nakachi et al. (2000)^10^	Japan (Saitama)	140	8,412	RR (95%CI) according to no. of cups/d		Protective
				<4	1.00(Reference)	NS
				4-9	Not reported	
				10+	0.69(0.23-1.88)	

Tsubono et al. (2001)^11^	Japan (Miyagi)	419	25,892	RR (95%CI) according to no. of cups/d		No association
				<1	1.0(Reference)	
				1-2	1.1(0.8-1.6)	
				3-4	1.0(0.7-1.4)	
				5+	1.2(0.9-1.6)	
				Trend R = 0.13		

Nagano et al. (2001)^12^	Japan (Hiroshima, Nagasaki)	901	37,639	RR (95%CI) according to no. of times/d		No association
				0-1	1.0(Reference)	
				2-4	1.0(0.82-1.2)	
				5+	0.95(0.76-1.2)	
				Trend P = 0.56		

Hoshiyama et al. (2002)^13^	Japan (National)	Men 240 Women 119	30,130 42,362	RR (95%CI) according to no. of cups/d		No association
				<1	1.0(Reference)	
				1-2	1.6(0.9-2.9) for men, 1.1(0.5-2.5) for women	
				3-4	1.1(0.6-1.9) for men, 1.0(0.5-2.1) for women	
				5-9	1.1(0.6-1.9) for men, 0.8(0.4-1.6) for women	
				10+	1.0(0.5-2.0) for men, 0.7(0.3-2.0) for women	
				Trend P = 0.634 for men, 0.476 for women		

Sun, et al. (2002)^14^	China (Shanghai)	190	563	RR (95%CI) for urinary epigallocatechin (mg/g creatinine)		No association**
				Negative	1.00(Reference)	
				<0.197	0.64(0.37-1.09)	
				0.197+	0.82(0.49-1.38)	
				Trend P = 0.77		

* NS, not significant. RR, relative risk. CI, confidence interval.

* Significant protective assoociations for subgroups of subjects with 4+ years of follow-up or lower levels of serum carotenes.

Our paper, entitled “Green tea and the risk of gastric cancer in Japan” was published in the New England Journal of Medicine in 2001.^11^ In this study, 26,311 residents of Miyagi Prefecture were followed for 9 years with the use of a regional cancer registry. We concluded that the risk of gastric cancer did not decrease even with high levels of green tea consumption. Although the study presented negative data, it was appreciated and published in the journal because it was a prospective cohort study that followed a large population for a long period and used very reliable data from a regional cancer registry.

## Does *β*-carotene prevent lung cancer and gastric cancer?

Let me introduce you now to epidemiologic studies on the relevance of *β*-carotene. The effects of green and yellow vegetables in preventing cancer have been suggested in numerous epidemiologic studies.

In 1965, Hirayama and co-workers started a large-scale prospective study. They followed up 265,118 residents aged 40 years or older from six prefectures of Japan for 17 years to investigate their lifestyles. Among males, the risk of death from all causes, from all cancers, and from lung cancer was higher in the group of people who did not eat green and yellow vegetables very frequently, as compared with that in the group of people who ate green and yellow vegetables every day (relative risk [RR] = 1.38, 1.32, and 1.28, respectively). Among females, on the other hand, a similar tendency (RR = 1.22) was seen for death from all cancers, but not for death from lung cancer (RR = 0.87).^15^

An ecological study was conducted by Tsubono and co-workers in five areas of Japan in which the mortality rates from gastric cancer differed by three folds among the areas. A total of 634 males aged between 40 and 49 years under the jurisdiction of each public health center were randomly selected, and the frequency of consumption of green and yellow vegetables by these subjects as well as by the wives of 373 men was investigated to determine the correlation. A strong negative correlation (*r* = -0.88) was observed between the mean frequency of consumption of green and yellow vegetables in each area and the mortality rate from gastric cancer. Moreover, the mortality rate from gastric cancer in males tended to be low (*r* = -0.31) in the areas in which the plasma concentrations of *β*-carotene were high.^16^^,^^17^

A great trend in epidemiologic studies began about 10 years ago. Cancer chemoprevention trials were started in several countries. These trials attempted to administer the substances that may inhibit the growth of cancer to a human population for the purpose of evaluating the degree of cancer prevention by these substances, that have been identified in epidemiologic studies and animal experiments. These studies were conducted as randomized controlled trials (RCTs) in large human populations. This study technique was employed because it is believed to be the most scientific method for directly demonstrating the preventive effects of the substances on the growth of cancer.

This study trend then spread worldwide. In 1982, the Physicians’ Health Study was started in the United States.^18^ In about 1985, the *α*-tocopherol and *β*-carotene (ATBC) Trial in Finland,^19^ and the Linxian Study in China^20^ were started. In 1988 and thereafter, the *β*-carotene and retinol efficacy trial (CARET) study^21^ and the Women’s Health Study^22^ were carried out. All of these trials are large RCTs conducted on a target sample of 20,000 to 40,000 people. Such studies were also designed to administer *β*-carotene, because of the widespread belief that it could prevent cancer. Only the study in China, which has since been completed, clearly demonstrated that intake of *β*-carotene reduced mortality rates from all cancers, including gastric cancer.^20^

## Results of the CARET study (United States)

In January 1996, the NIH of the United States announced in a press release the discontinuation of *β*-carotene and vitamin A administration for lung cancer prevention in their CARET study. The reason was that the results of an interim analysis after administration of these substances for 4.5 years on average unexpectedly indicated a 28% increase in lung cancer incidence in the group given *β*-carotene. Administration of *β*-carotene was also immediately discontinued in other ongoing chemoprevention trials. In May 1996, a paper on this event was published in the New England Journal of Medicine.^18^^,^^21^ This news created a great sensation among epidemiologists and cancer researchers all over the world, and we learned three important lessons.

One of these lessons was “success in failure.” The study itself was a failure because the results were entirely unexpected. However, the apparent lack of efficacy of *β*-carotene, which had until then been believed to be an effective component, was proven as scientifically correct knowledge. If this study had not been conducted and administration of *β*-carotene had been continued, much injury to health could have been caused all over the world, and a tremendous amount of money would have been wasted during the subsequent decades. We can say that the negative data helped in preventing such tremendous loss.

The second lesson was re-appreciation of the basis of public health activities, namely, recognition of the value of eating vegetables. Green and yellow vegetables contain, besides *β*-carotene, numerous carotenoids, such as *α*-carotene, lutein, and lycopene. There are various possible reasons for the failure of the intervention study, such as the amount of *β*-carotene being too large, or the synthetic substances used being different from the corresponding natural substances. However, green and yellow vegetables are still useful for preventing cancer. *β*-carotene might be a mere marker of green and yellow vegetables.

The third lesson is the importance of RCTs in human populations. The effect of *β*-carotene in cancer prevention has been suggested by the results of animal experiments. However, it was not proved in human. Findings in animal experimentation are not always true in human beings. Evidence for health and diseases in human has to be sought for observation and intervention studies for human. We learned the importance of verification in human populations.

The US NIH acted promptly after obtaining the interim report of the intervention study. NIH authorities announced that “intake of *β*-carotene from sources other than food is not recommended,” “consumption of vegetables and abstinence from smoking are the best ways to prevent cancer,” and “discontinuation of clinical studies using *β*-carotene is recommended.” It is unknown whether *β*-carotene plays an essential or partial role in the prevention of cancer, or it is just a marker. However, the results of this study provoked many suggestions at a time when cancer chemoprevention trials began to attract attention in the field of preventive medicine. It is not easy to identify the truth or the precise factors affecting diseases.

## Scientific and ethical aspects of studies targeting humans

How have medications that we are now taking been found to be “effective”? RCTs are considered to be the best method, because the subjects are randomly assigned to an intervention group or a control group. RCTs are easy to run in animal experiments, but not so in human ones. Healthy people or ordinary patients are the subjects of such trials, so how many people would truly want to cooperate with studies for drugs of unknown effects and submit themselves to random allocation to the intervention or the control group and take a drug without knowing what they are taking? Recently, the importance of informed consent has been emphasized and people often say that the choice of the patients and their family should be respected as a general rule. But can we really do that?

When a highly scientific method of investigation is applied to humans, the ethical aspects of the study also become an issue. The more the ethical aspects are respected, the less scientific the study tends to become. However, it would also be unethical to use new drugs and vaccines without scientific evidence. The ethical aspects and the scientific aspects are thus both very important, but conflict with each other, often causing dilemmas.

## What makes a study “scientific”?

Whether or not an epidemiologic study is “scientific” depends on the choice of study design in maximizing validity and reliability. In terms of reliability or reproducibility, in particular, epidemiologic studies face the limitation that the studies target humans. Therefore, the multiple studies in different countries and regions by different researchers should be obtained. Meta-analyses are sometimes necessary.

In studies targeting humans, ethical problems usually arise if an experimental technique that improves scientific validity is chosen. RCTs are considered to have the best validity for studies in humans.

Why is this technique scientific? You will probably be able to understand the reason easily if you think of the fact that, in the past, the logic of the three “did’s” was used to present evidence. The logic of the three “did’s” was, “we administered the drug, the patient was cured, therefore, it worked,” although no control group was used. Even if a drug is administered and the patient is cured, it is impossible to draw the conclusion that the drug works. It might have looked as though it worked, owing to a placebo effect. The action of the doctor’s other instructions — rather than taking the drug itself — might have worked. Or simply a change of exercise and diet habits might have worked. The logic based on the three “did’s” without a control group is inadequate to relate the effects to the drug. How difficult experiments designed to obtain scientific evidence in humans can be! Moreover, things are by no means easy when ethical aspects are also taken into consideration. This is true of not only clinical studies, but also of epidemiologic studies.

## Bias

In epidemiology, biases are defined as deviations from the true values in one direction (systematic error). The word has two meanings, namely, biases in the determined values and biases in the observed populations.

There are numerous kinds of bias. Of the various kinds of bias, four major biases impair the validity of clinical and epidemiologic studies. They are sampling bias, selection bias, measurement bias, and confounding bias. Sampling bias affects the external validity. In other words, it affects the universality or generalization of the study results. For example, opinion polls are trusted as far as they represent the general population on the basis of a precondition that sampling bias is not present. The remaining three biases affect the accuracy of the study itself, in other words, the internal validity. Among them, selection bias and measurement bias can be eliminated during the process of formulation of the study design before the start of the study. However, the last confounding bias should be carefully examined at the time of the analysis and interpretation of the study data. In many cases, multivariate analysis is used to solve this problem. Whether an epidemiologic study becomes scientific or not depends on how we can minimize these biases.

## Scientific validity of clinical and epidemiologic study techniques

Of the various clinical and epidemiologic study techniques, RCTs are ranked at the top in terms of scientific validity. They are a kind of experiment using humans, and are also referred to as interventional studies. For the Cochrane Project that is conducted to collect the basic data for evidence-based medicine (EBM), only RCTs conducted throughout the world are collected and analyzed.^23^

RCTs are not as easy as they may look. No matter how scientific and valid a research technique might be, there are always problems of cost, duration, finding people who will agree to participate in the study, and ethical issues.

When an RCT is to be conducted, judgment should first be made as to whether the research subject warrants an RCT. RCTs should not be conducted, for example, for evaluating the most advanced organ transplantation technology without which patients may die, or in cases where a new technology is in the very early stage of development. Moreover, RCTs are unnecessary if long-term clinical experience has clearly demonstrated the efficacy of the treatment, even if the efficacy has not been proved in an RCT. One example is that of digitalis.

Second, the feasibility of conducting an RCT should be examined beforehand from the viewpoint of the expected sample size, study duration, cost, and administration. In some cases, a feasibility study may be necessary.

The third consideration is the most important, namely, the potential ethical problems of the study. Because humans are used as the subjects, strict inspection of the validity of the randomization of patients or normal healthy subjects and validity of the method of obtaining informed consent is required.

RCTs conducted in humans are almost always associated with problems of compliance and contamination. Let me give you two examples. One was a study of annual changes in compliance with screening in an RCT for evaluating the efficacy of gastric cancer screening.^24^ In this study, we enrolled patients from several towns in Miyagi Prefecture to evaluate the efficacy of gastric cancer screening, and we then examined the variations in compliance with screening by an RCT using group randomization. The 50-year-old and 60-year-old men who were sent a reminder to undergo a gastric cancer screening test by direct mail from the town mayor showed higher compliance in the first year than did those in the control group, who did not receive the letter. From the next year on, however, subjects aged 60 years or older in the control group showed higher compliance. This means that the compliance in the group that received the reminder decreased, and contamination occurred in the control group. Accurate evaluation was impossible in such a situation. We had to discontinue the study prematurely.

A similar event took place in the famous Mayo Lung Project conducted in the 1970s to evaluate the efficacy of lung cancer screening.^25^ This study failed to demonstrate the efficacy of lung cancer screening, but a decrease in compliance was found in 25% of the patients who were reminded to undergo lung cancer screening (intervention group) and contamination was noted in 55% of those who were not particularly reminded to undergo screening (control group). One out of two persons in the control group were found to have taken the screening test secretly. When an interventional study cannot be conducted precisely according to the protocol owing to low compliance and high contamination, careful attention must be paid in evaluating the study results. The results may suggest a lack of efficacy, whereas in truth the screening is effective, or they may suggest only small efficacy when the actual efficacy is great.

## Criteria for judgment of ethical aspects

Interventional studies by RCT with great scientific validity are associated with ethical problems. In the case of clinical studies of patients, the famous four great rules of Beauchamp and Childress apply as the criteria for judgment of the ethical aspects.^26^ They are to confer benefits, to bring no harm, to respect the patient’s right to decide for him or herself, and to equally share the benefit and loss.

However, when it comes to consideration of ethical aspects in studies, medical ethics in Japan are not quite the same as those in the rest of the world, even if these four great rules, which appear to reflect the universal sense of value of human beings, are abided by. Ethics may differ, depending on religion, race, region, national history, level of scientific technology, and the economic situation. Something that is considered “good” in Japan may be considered “not good” in another country, or vice versa. You will probably be able to understand this more easily if you think about differences among different countries in attitudes toward organ transplantation based on brain death.

## Informed consent, protection of personal information, and public benefit

Probably almost everybody knows the words, “informed consent.” The seeking of informed consent shows both recognition that patients and examinees should play leading roles in medical care and health examinations and respect for their rights to decide for themselves. In addition, the seeking of informed consent can be expected to improve the quality of medical care and health examination, thereby improving its efficacy and safety. The patients and examinees can choose the method they prefer. The last thing, but not the least, is that informed consent promotes a trusting relationship between the patient and the doctor, or between the examinee and the examiner.

In recent years, epidemiologic and clinical studies involving the analysis of human and viral genes have often been discussed by the mass media. There are instances of the mass media reporting on studies that were conducted with informed consent but failed to take appropriate measures to keep up with the rapid progress of the gene analysis technology.

The more advanced medical technology becomes, the more important the approach becomes, from the viewpoint of social medicine. The conflict between disclosure of information in medical settings, personal rights according to the laws protecting personal information, and important public benefit in such fields as predictive medicine becomes more and more intense.

## Changing epidemiology into a “neotype science” and transformation of EKIGAKU (epidemiology) into EKIGAKU (beneficial science)

Academic research does not come from any specific necessities, but arises from the intellectual curiosity of scientists or their desire to break new ground. This paradigm has long been accepted, and education is provided to stimulate this style of research. The older general universities in Japan have traditionally recognized and fostered such research and researchers.

In recent years, however, due to global scientific and technological competition and changes in the national financial scale, many research prrograms have become bigger, necessitating external evaluation, and have been transformed into studies of the goal-achieving type. Yoichiro Murakami called this a “neotype science” and described the conventional approach as “prototype science.” The Millennium Project and the Medical Frontier Strategy on which the Japanese Government is about to focus its efforts are actually heading in the direction of neotype science. In neotype science, not only a group of specialists in science, but also members of other organizations or institutions, or even taxpayers in some cases, may evaluate the research. Obviously, it is then necessary for the research to become ever more scientific and ethical.

Because of recent advancements in research methodology, classical epidemiology has undergone a dramatic transformation in terms of its scientific nature and need for evidence-based evaluation. In modern epidemiology, which requires large-scale research organizations, tremendous cost and prolonged study period, the mere intellectual curiosity of scientists cannot be accepted as motivation. The need to transform EKIGAKU (epidemiology) into EKIGAKU (beneficial science) will always be important.
